# Aortic and left pulmonary artery dilatation: an unusual cause of a left hilar mass and lung collapse

**DOI:** 10.11604/pamj.2014.18.130.2993

**Published:** 2014-06-10

**Authors:** Adenike Temitayo Adeniji-Sofoluwe, Atiku Hafiz

**Affiliations:** 1 University of Ibadan, College of Medicine, Department of Radiology & University College Hospital, Ibadan, Nigeria

**Keywords:** Pulmonary artery dilatation, lung collapse, aorta

## Image in medicine

An 80 year old clergy, presented with a 5-day history of left-sided chest pain which was non-radiating but had cough productive of white sputum. No history of haemoptysis, dyspnoea, orthopnoea or paroxysmal nocturnal dyspnoea was elicited. However, a history of smoking 2 cigarettes per day for 10 years was present. He is a known hypertensive on medications,also being managed for diverticulosis and Benign Prostatic Hypertrophy. Respiratory rate was 26cycles per minute with crepitations in the left mid-and lower lung zones. Bilateral pedal oedema was found but the cardiovascular and gastrointestinal systems were normal. Requests for an ultrasound, a chest X-rayCXR and Contrast Enhanced computerised tomography CECT of the chest were made. High resolution CECT of the chest was performed with a 64-slice Toshiba CT. Multiplanar images in axial, coronal and sagittal planes were acquired at 1mm cuts in pre-and post-contrast series. Images depicting the abnormality are shown (A, B, C,D). No further invasive workup like a biopsy was indicated following imaging. Prior to the CECT, bronchogenic CA was considered in view of the long standing smoking history. The role of Computerised tomography in the evaluation of chest symptoms has been emphasised in these images.

**Figure 1:**
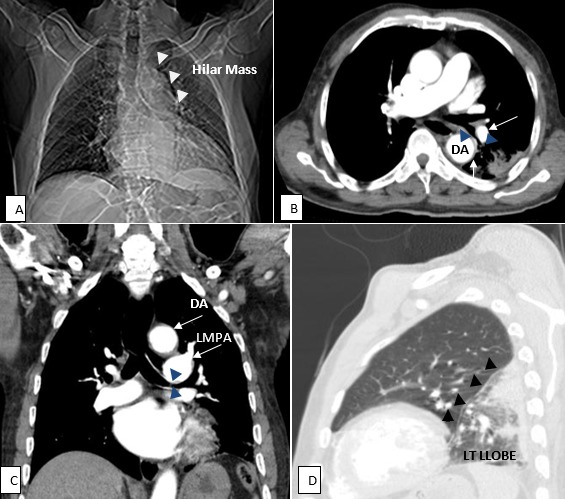
Contrast Enhanced Computerized Tomography of the chest. A) The Scanogram showed haziness of the left hemi thorax with a lobulated soft tissue mass (White Arrow heads) in the left hilar region; B) Axial view in mediastinum window confirm the mass to be an enlarged left main pulmonary artery LMPA and its branches (White arrows) as well as an ectatic descending aorta DA [luminal diameter-5.1cm]; C) Coronal view in mediastinum window shows associated compression of the left main bronchus (Blue Arrow heads).; D) Sagittal View in lung window shows resultant consolidation-collapse of the left lower lobe sparing only the apical segment. (Black Arrow heads)

